# Reorganization of Functional Networks in Mild Cognitive Impairment

**DOI:** 10.1371/journal.pone.0019584

**Published:** 2011-05-23

**Authors:** Javier M. Buldú, Ricardo Bajo, Fernando Maestú, Nazareth Castellanos, Inmaculada Leyva, Pablo Gil, Irene Sendiña-Nadal, Juan A. Almendral, Angel Nevado, Francisco del-Pozo, Stefano Boccaletti

**Affiliations:** 1 Complex Systems Group, Universidad Rey Juan Carlos, Fuenlabrada, Spain; 2 Laboratory of Biological Networks, Centre for Biomedical Technology, Madrid, Spain; 3 Cognitive and Computational Neuroscience Lab, Centre for Biomedical Technology, Polytechnic and Complutense University of Madrid (UPM-UCM), Madrid, Spain; 4 Memory Unit, Hospital Clínico San Carlos, Madrid, Spain; 5 Computational Systems Biology Group, Centre for Biomedical Technology, Madrid, Spain; 6 Istituto dei Sistemi Complessi, CNR, Florence, Italy; University of Michigan, United States of America

## Abstract

Whether the balance between integration and segregation of information in the brain is damaged in Mild Cognitive Impairment (MCI) subjects is still a matter of debate. Here we characterize the functional network architecture of MCI subjects by means of complex networks analysis. Magnetoencephalograms (MEG) time series obtained during a memory task were evaluated by synchronization likelihood (SL), to quantify the statistical dependence between MEG signals and to obtain the functional networks. Graphs from MCI subjects show an enhancement of the strength of connections, together with an increase in the outreach parameter, suggesting that memory processing in MCI subjects is associated with higher energy expenditure and a tendency toward random structure, which breaks the balance between integration and segregation. All features are reproduced by an evolutionary network model that simulates the degenerative process of a healthy functional network to that associated with MCI. Due to the high rate of conversion from MCI to Alzheimer Disease (AD), these results show that the analysis of functional networks could be an appropriate tool for the early detection of both MCI and AD.

## Introduction

A key issue in neuroscience is the understanding of the coexistence of local specialization and long distance integration in the complex structure of the brain. Graph theory provides valuable tools to describe the topological organization supporting cognitive processes [1]. In particular, the approach led to a characterization of structural and functional networks in the brain [2]–[4], typically endowed with high clustering and short non-Euclidean distance between nodes, the fingerprint of a Small World (SW) architecture [5]. In addition, graph analysis may help to identify network signatures of impairment in pathological conditions, such as the network organization in Alzheimer's Disease (AD) [6]. AD, the most frequent cause of dementia, is characterized by accumulation of beta-amyloid proteins, degeneration of neurons, loss of synaptic contacts, and it has been described as a disconnection syndrome [7]. Stam *et al.* [6] demonstrated that functional networks of AD patients show a loss of SW properties [6], [8], [9], resulting in an increase in the mean path length between nodes [8], with an associated decrease in local synchrony [9]. A crucial point is whether the pathophysiology of AD would be detected long before the actual diagnosis of the disease [10]. Indeed, the identification of preclinical AD could significantly enhance the benefit of new drugs and vaccines, at the time when the severe brain damage, such as widespread brain atrophy, associated with AD, has not taken place yet.

On the other side, Mild Cognitive Impairment (MCI) is an intermediate state between healthy aging and dementia [11]. In fact, 12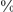 to 15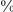 of MCI subjects develop some form of dementia per year. This makes MCI patients an ideal population to search for neurophysiological profiles of prediction of who will develop dementia. In amnestic MCI, cognitive abilities are mildly impaired, and patients are able to carry out everyday activities, but there are pronounced deficits in memory tasks. Whether MCI subjects show a similar network profile than AD patients is still a matter of debate. Neuropathological studies indicate that MCI patients share some of the AD pathophysiological characteristics, such as the presence of neurofibrillary tangles, loss of dendritic spines and the accumulation of beta-amyloid protein in the associative cortex [12]. fMRI studies show higher blood flow values in medial temporal lobe regions during a memory task in MCI, as compared to controls [13]. Bajo et al. [14] described higher functional connectivity values from MEG recordings in MCI subjects than in age-matched controls.

To our best knowledge, no previous characterizations of the topological properties of functional brain networks in MCI subjects with MEG were attempted so far. We here apply methods from complex networks theory to compute macroscopic and mesoscopic parameters of the functional networks in a group of nineteen MCI patients and a group of control participants of the same size. Brain activity was measured by means of MEG during a Sternberg's letter-probe memory task [15], [16] and functional connectivity was calculated using the synchronization likelihood (SL), a measure to evaluate the generalized synchronization based on the theory of nonlinear dynamical systems [17]. We will show that an increase in global network synchronization in MCI patients occurs, as compared to healthy controls, and that an evolution of the MCI functional network towards a more random structure takes place. Interestingly, MCI patients feature an increased synchronization between brain areas [14], and AD patients a corresponding decrease in connectivity [18]. Finally, based on the experimental observations, we offer a computational evolutionary network model that simulates the transition from healthy to MCI topology, and satisfactorily reproduces the changes in the network metrics observed in MCI subjects.

## Materials and Methods

### Data

MEG scans were obtained from nineteen MCI patients and nineteen healthy volunteers during a Sternberg's letter-probe task (see *Materials and Methods* in File S1 for details). Before the MEG recordings, all participants or legal representatives gave written consent to participate in the study, which was approved by the local ethics committee of the Hospital Clnico San Carlos. Data segments free of artifacts corresponding to eye blinks, eye movements of muscular activity were chosen by visual inspection. Five frequency bands [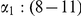 Hz, 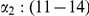 Hz, 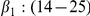 Hz, 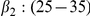 Hz, 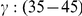 Hz] were considered. Synchronization Likelihood (SL) [17] was calculated between all channel pairs for each frequency band. A normalization was applied to obtain a probability matrix from which the topological network parameters are extracted. In what follows we define the normalization method and the metrics calculated over all networks.

**Figure 1:**
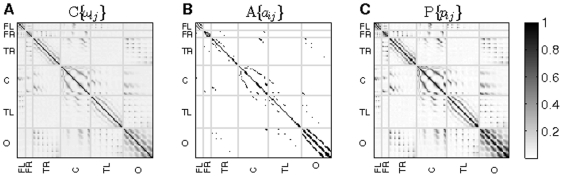
Functional network projection. Functional networks from a representative control volunteer. A broad-band filter was applied. (A) Weighted SL matrix obtained from the SL between 148 sensors. (B) Unweighted adjacency network after converting the SL matrix 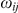 (shown in A) into a binary matrix using as a threshold 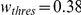, which leaves the 5% of all possible links. (C) Probability matrix after normalizing 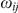 as explained in the text (note the contrast enhancement). In all panels, nodes/sensors are grouped according to the lobe they belong to: frontal left (FL), frontal right (FR), temporal right (TR), central (C), temporal left (TL) and occipital (O).

The SL between the 148 sensors yields a (symmetric and weighted) 148
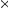
148 correlation matrix 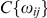. The values of the matrix elements range from 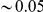 to 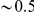, which corresponds to a difference of one order of magnitude between the maxima and the minima. The matrix is fully connected, and all pairs of nodes (sensors) have a SL higher than zero. Traditionally, two different techniques are used in order to study weighted brain networks. The first method involves thresholding the matrix to obtain an unweighted network 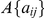, so that the link between node 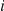 an 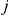 is 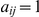 if the weight of the connection is above the threshold, and 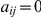 otherwise. In some other occasions, a fraction of the total number of links is kept [19] (e.g., the 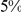 of the highest weighted links). In both cases, information is lost by thresholding. Our approach relies in a normalization technique recently proposed [20] that allows using the measures applied to unweighted networks to the weighted case without losing the information contained in the weights distribution. In addition, this normalization facilitates comparison between networks obtained from different individuals. By mapping the weights of the correlation matrix 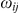 with a continuous bijective map 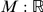 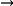
[0,1] it is possible to obtain a probability matrix 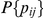. In our case, we linearly normalize the weights 
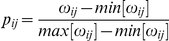
. The matrix 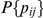 reflects the probability of existence of a link between node 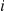 and 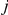, and an ensemble of unweighted matrices can be generated on the basis of the probabilities given by 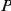. The power of the approach is that any polynomial function calculated as the average of an ensemble of adjacency matrices obtained from 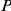, is equal to the value of the polynomial of the matrix 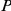 itself [20]. Therefore, one can extend several classical measures for unweighted networks to 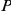. To visualize the advantage of this method, we have plotted in Fig. 1 the matrices 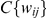, 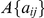 (with 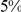 of the links) and 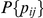 for a control individual, grouping nodes according to the lobe they are over. We can see that in the case of the adjacency matrix, Fig. 1*B*, we lose information, which is specially relevant for the inter-lobe correlations (e.g., see connections between central and occipital lobe). In addition, by comparing 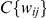 and 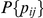, we observe how the matrix normalization enhances the contrast between low and high correlated nodes.

### Definition of network parameters

As for the network parameters, the average degree of a node 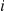 is obtained as 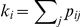, and the mean degree 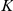 is 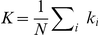. The mean shortest path 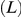 can be obtained as follows: the length 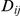 associated to the link connecting nodes 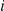 and 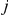 is defined as the inverse of its probability 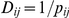, being 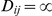 when 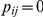. By applying the Dijkstra's algorithm [21], the shortest distance matrix 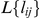 is found. The value 
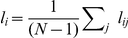
 tells us how far is node 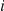 from the rest of the network, while the average 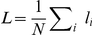 gives the average shortest path of the whole network. The mean clustering 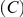 reflects the probability of finding triangles in the network. It can be calculated through the probability matrix as 
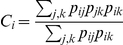
. The average clustering coefficient is obtained by averaging 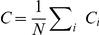 [20].

The node outreach 
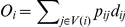
 relates the distance and the weight of the connections of node 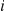, being 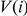 the set of nearest neighbors of node 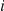 and 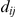 the physical (Euclidean) distance of the links (obtained from the distance between sensors). The network mean outreach 
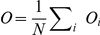
 reflects whether the network activity is dominated by short-range (low outreach) or long-range (high outreach) connections. Finally, the network modularity 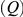 quantifies the existence of topological communities inside the network [22]. Its value is 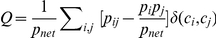, where 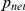 is the sum of all terms of 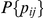, 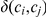 is the Kronecker delta and 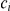 and 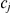 are the communities of nodes 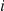 and 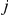, respectively. In what follows, we focus on assuming the classical network partition into six lobes (central, frontal-left, frontal-right, temporal-left, temporal-right and occipital).

In order to evaluate the deviation of the network parameters from their corresponding randomized versions, we have generated 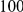 network surrogates by randomly permuting the coefficients of the matrix 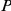. Finally, we have normalized the metrics with the average of the set of surrogate matrices, 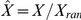.

## Results

### Network structure and global properties

For each individual, we construct a probability matrix from the broadband signal 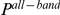 and five probability matrices from each considered frequency band 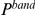 (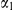, 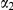, 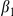, 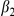 and 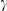). Next, we compute the network parameters described in the previous section and average them by groups (control and MCI). File S1 summarizes the results obtained for each group along with the percentage of variation from the control group. The average degree of the network 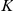 shows an increase of 15.9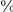 for the MCI group. Since only positive recognition trials during the memory paradigm are considered, these results confirm that MCI patients require higher synchronization in their functional networks in order to perform a memory task [14]. We also observe that differences between both groups are more evident in the broadband signal, a signature that will be constantly present for all network parameters. As a consequence of the higher number of connections in the MCI group, the average shortest path 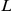 decreases, although differences between both groups are less significant. It is interesting to note that the normalized shortest path 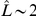 in both controls and MCI, revealing that the average distance between nodes is twice as large as for an equivalent random graph. Since 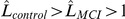, the organization of the shortest paths within the MCI network is slightly shifted towards more random configurations.

The outreach parameter 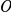 is the most affected parameter. We observe a 23.4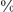 increase for the broadband signal, which is higher than the 15.9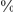 increase in mean degree for both networks. This indicates that the increase in correlation between nodes in the MCI networks becomes more pronounced at long-range connections, and the combination of both alterations makes the outreach parameter the one with the highest differences between both groups. This suggests that individuals suffering from MCI incur in a higher energetic cost than controls to perform the same memory task, since they have to maintain high correlations at longer distances. The normalized outreach 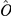 is in both cases lower than in the random case (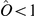) since the existing correlations between nearby brain regions are spread around the whole network when randomizing it. Nevertheless, we observe that the MCI group has a 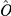 closer to one, which again reveals that the functional structure is more random than in the control group. Finally, there is a decrease in the modularity 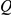 that is in accordance with an evolution towards random topologies. This reduction of 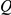 in the MCI group, larger again for the broadband signal, indicates a degradation of the modular structure of the functional networks, and it is an inherent property of random networks, whose modularity is close to zero.

**Figure 2:**
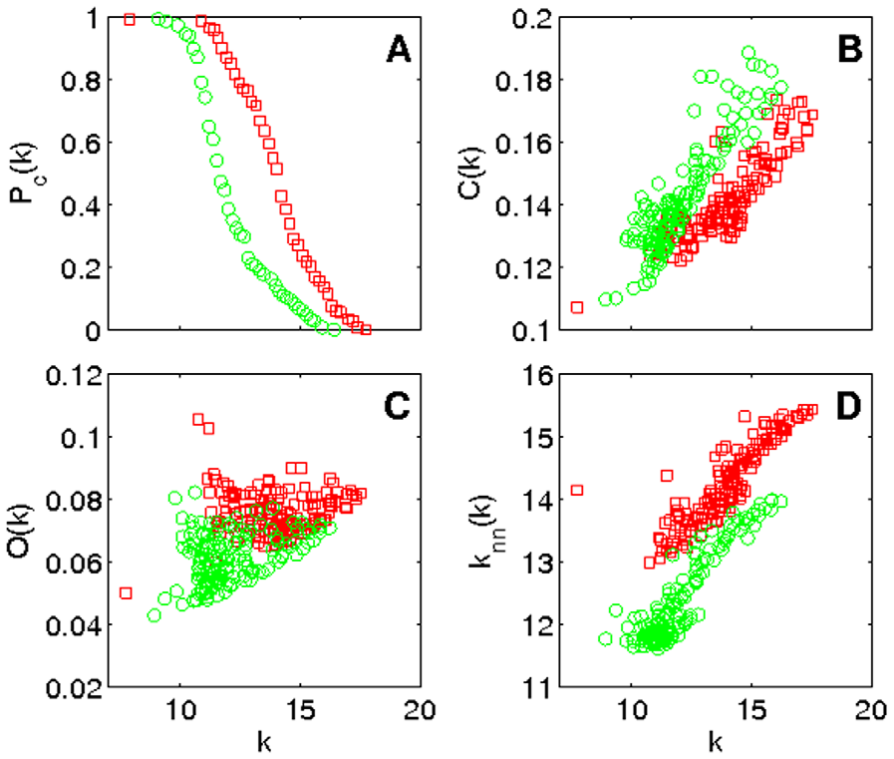
Network parameter distributions. Several network parameter distributions for the control (green circles) and MCI (red squares) groups. (A) Probability distribution of finding a node with a degree higher than 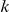, (B) clustering coefficient 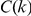, (C) outreach 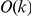 and (D) average nearest neighbors degree 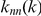.

Figure 2 shows the behavior of the degree distribution, clustering, outreach and neighbour's mean degree – as a function of the node average degree 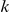 for control (green circles) and MCI groups (red squares) computed from the broadband signal. In Fig. 2(*A*) we report the cumulative degree distribution 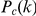 which, in turn, corresponds to the average degree of an ensemble of unweighted networks generated using the probability matrix. The figure makes it evident the likelihood of finding highly connected nodes within the MCI group. As for the clustering distribution 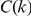, both groups have positive correlations (see [Fig. 2(*B*)]), a behaviour that has been previously reported in healthy individuals and Alzheimer patients [8]. Notice that individuals suffering from MCI have lower clustering coefficient, entailing an evolution towards random structures, where the number of triangles is much lower than in the networks analyzed here [2]. The outreach distribution 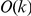 [Fig. 2(*C*)] shows that the MCI group features higher values of the outreach. Since 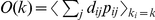 (where 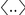 indicates ensembles average), the latter feature comes from an increase in the probabilities of long distant links. In other words, the evolution of the disease has, somehow, increased the weight of long-range connections. Finally, in Fig. 2(*D*) we report the average degree of the nearest neighbours of nodes with degree 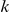, 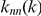. This distribution characterizes the assortativity of the network [23]. Both groups show a positive degree correlation, revealing the assortative nature of the networks. Interestingly, assortative organization has been already reported in functional connectivity networks obtained with fMRI [24]. Despite both networks being assortative, the MCI group exhibits higher 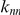 values, as a result of the much larger levels of synchronization between nodes.

To compare the mentioned network parameters between the two groups, each parameter value was first averaged across epochs for each participant and channel pair. Then, nonparametric permutation testing [25]–[27] was applied to find channel pairs with significant differences between groups. In brief, a two-sample non-parametric test (Kruskal-Wallis test) between groups was performed. Next, non-parametric permutations were calculated by randomly dividing the 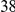 participants into 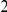 groups of 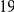 members to match the numbers in the original groups. This was repeated 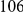 times for each channel pair. Subsequently, the threshold was obtained from the 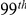 percentile of this set of 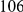 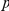-values. After the application of this statistical method to SL raw data (i.e., without band-pass filtering) there are 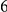 parameters showing significant differences between the two groups: outreach 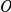 (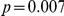), normalized clustering 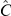 (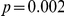), modularity 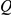 (p = 0.0033), mean degree 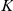 (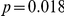), normalized shortest path 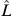 (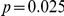) and normalized outreach 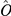 (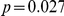) (see File S1 for details).

### Mesoscale analysis: inter-lobe communication, community structure and roles

From a holistic point of view, it is well known that the processing abilities of the brain rely on the segregation and integration of information [28]. Since both mechanisms depend on the modular structure of the network, any alteration of the interplay between the existing clusters may lead to a deterioration of the functional network performance. With the aim of evaluating how MCI modifies the modular structure, we have measured the internal lobe strength 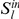, the external lobe strength 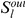 and the lobe modularity 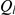, being 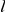 the lobe index. The two former parameters measure, respectively, the total weight of the connections inside lobe 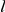,
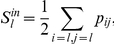
and those going to other lobes 
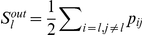
.

**Figure 3:**
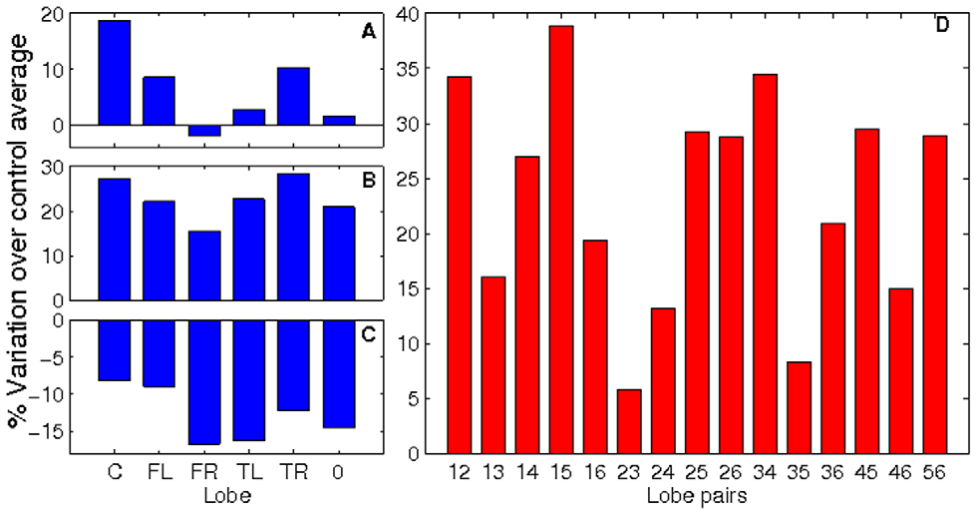
Mesoscale analysis. Percentages of variation in the MCI group with respect to the control one of: the strength inside each lobe (A), the strength of the links going out from each lobe (B), and the lobe modularity (C). In (D), percentages of variation of the lobe-to-lobe strength. Lobe code: 1 = central, 2 = frontal left, 3 = frontal right, 4 = temporal left, 5 = temporal right and 6 = occipital.

Figure 3 summarizes the variation of these parameters in the MCI group for the classical cortical division into six lobes (central, frontal left, frontal right, temporal left, temporal right and occipital). With regard to the internal lobe strength [Fig. 3(*A*)], we can see that three lobes have a significant increase of their internal activity, specifically, the central (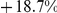), the frontal left (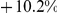) and the temporal right (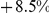), and only the frontal right lobe has slightly reduced its internal synchronization (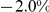). Differences in the external lobe strength are more important [Fig. 3(*B*)], with an increase higher than 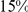 in all lobes, indicating that, besides an evolution towards random structures, there is an increase in the weight of the connections between lobes in MCI. As a consequence, the modularity of all lobes decreases [Fig. 3(*C*)], since the restructuring of the network is dominated by the increase of the inter-lobe connections. Therefore, despite the increase in communication between lobes, the segregated structure of the brain is dramatically reduced and the balance between segregation and integration present in a healthy brain is lost. Finally, we have plotted the percentage of variation of the lobe-to-lobe strength [Fig. 3(*D*)], which shows in all cases a positive value.

**Figure 4:**
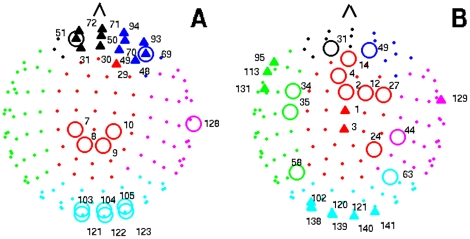
Community structure and roles. (A) Nodes with higher within-module degree 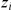 and participation coefficient 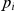 in healthy individuals. Only the first 13 nodes with the highest 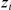 and 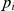 are labelled. Those with the highest 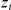 are marked with circles and triangles indicate those with the highest 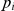. (B) Nodes with higher variation at the within-module degree and participation coefficient in the MCI group. Again, only the first 13 nodes with the highest differences are labelled: nodes with higher increase of 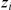 (circles) and 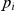 (triangles). Lobe color scheme: red (central), blue (frontal right), black (frontal left), magenta (temporal right), green (temporal left), and cyan (occipital).

Next, we have gone down to the lowest scale (i.e., the node level). We have used the classification of nodes introduced by Guimerà et al. [29], which is based in the computation of the within-module degree 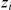 and the participation coefficient 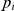. The first parameter, quantifies the importance of node 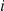 inside its community and it is defined as 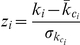, where 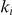 and 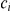 are, respectively, the degree and the community 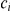 of the node 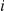, 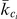 is the mean degree of the community and 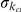 is the standard deviation of 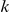 in 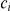. On the other hand, the participation coefficient 
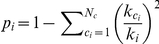
 indicates how connections of the node 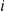 are distributed among the existing communities, where 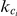 is the number of connections between node 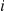 and community 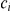 and 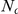 is the total number of communities. The participation coefficient is zero when all links of a node are inside its own community and close to one when they are distributed among all modules of the network. Figure 4(A) shows the position of the nodes with higher influence in their communities (circles) and higher participation coefficients (triangles) in the healthy group. We can observe that, during a memory task, most participating nodes are located over the two frontal lobes, while nodes with higher relevance (i.e., those with higher weights) are located over the occipital lobe. Figure 4(*B*) shows those nodes which have suffered the highest variation of both parameters in the MCI group. We observe a generalized increase of the participation coefficient, while the within-module degree has both positive and negative changes, which indicates that a certain reorganization is occurring inside each lobe. Note that nodes with higher increases in the participation coefficient are located over the occipital, temporal right and central lobes, while nodes for which the within-module degree has increased the most are spread over the whole network (see File S1 for more details).

### Modelling network changes: the emergence of MCI

**Figure 5:**
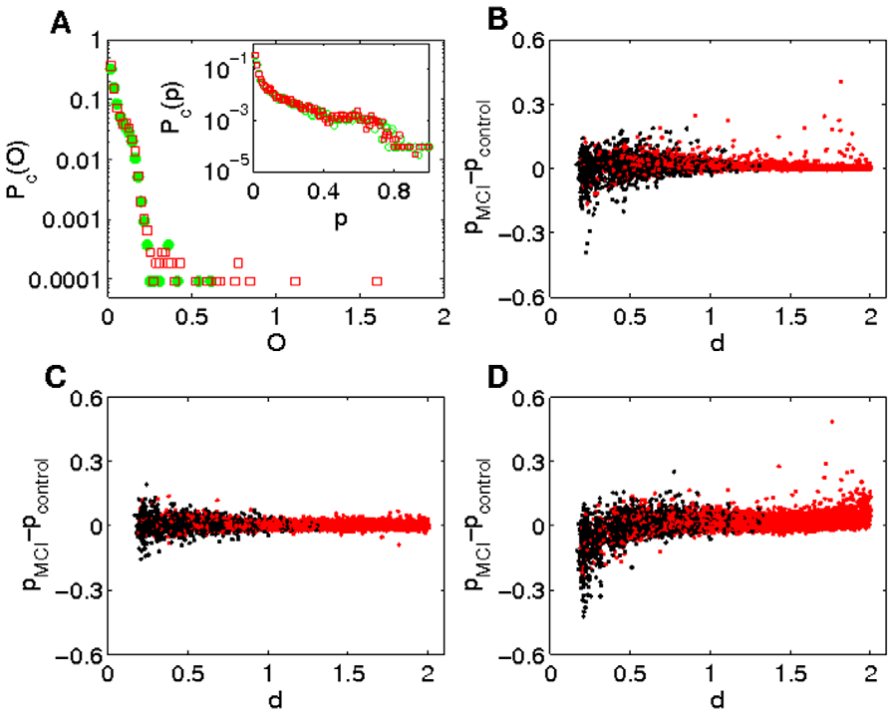
Relationship between lengths and weights. (A) Cumulative probability distribution of the normalized weights (inset) and outreach for the control (green circles) and MCI (red squares) group. Despite having similar weight distribution, links with high outreach coefficient are more probable for the MCI group. (B) Variation of the link weight (MCI minus control), black circles correspond to intra-lobe connections and red circles to inter-lobe ones. (C) Variation of the link weight obtained with the evolutionary model without considering the influence of the link length (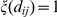). (D) Variation of the link weight considering the length influence. Parameters used in the simulations are: 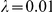, 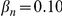, 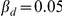 and 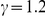.

All previous results indicate that mild cognitive impairment is related to a random increase in synchronization between brain areas. In order to model this phenomenon, it is necessary to understand how weights are distributed within the network, since the disease modifies the correlations between nodes. Figure 5(*A*) shows the probability 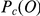 of finding a connection with an outreach coefficient higher than 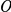 in the control (green circles) and MCI (red squares) groups and the inset plots report the probability 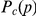 of having a link with a normalized weight higher than 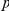. We highlight a power law scaling in the weight distribution, with a truncated tail in both groups, similar to what is observed in anatomical [30] and functional networks. In contrast, they do not share the same outreach distribution, since the probability of finding nodes with high outreach is higher in the MCI group. This discrepancy is a consequence of a shift of higher weights (i.e., correlations) to links with longer distances, increasing the outreach of the links. In order to confirm this observation, we plot in Fig. 5(*B*) the increase in the weight of each link (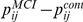) as a function of its length. Red and black circles correspond, respectively, to intra-lobe and inter-lobe connections. Despite the global strength is here higher in the MCI (since 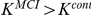), there are both positive and negative changes, so the increase of the correlation between nodes is not a generalized behavior. Nonetheless, there exists a number of long-range connections that significantly increase in weight while, at the same time, the weights of some short-range connection drastically decrease [see Fig. 5(*B*)]. This fact indicates that in MCI patients there is an increase in correlations at long distances and a decrease of short range connections.

Our model can be discussed as follows: a) we randomly select a link in the correlation network 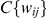, b) we modify the initial weight 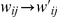, c) we obtain the new probability matrix 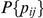 and recalculate all network parameters and d) we repeat sequentially the previous steps from a). The new values of 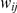 are bounded by the maximum and minimum of the initial correlation matrix. At each time step, the value of 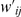 of the modified connection is obtained by the expression 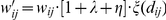, where 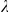 is the degradation rate, a constant related to the average increase of the network strength, 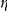 is a white noise term with zero mean and amplitude 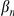, and 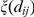 is a function that introduces the influence of the length of the link. Figure 5(*C*) and (*D*) shows two numerical simulations obtained with 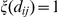 (i.e., no influence of the length) and 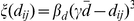, where 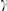 regulates the influence of the distance to the average length 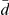 and 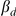 is the amplitude of the length dependency. We can see how, in both cases, the model successfully reproduces the bell-shaped behavior of the weight variation. Nevertheless, a length-dependent term 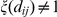 has to be included to account for the increase in long-range connections and the decrease at short distances. In the example plotted, a cubic function is chosen, but the adequate function is still an open question.

**Figure 6:**
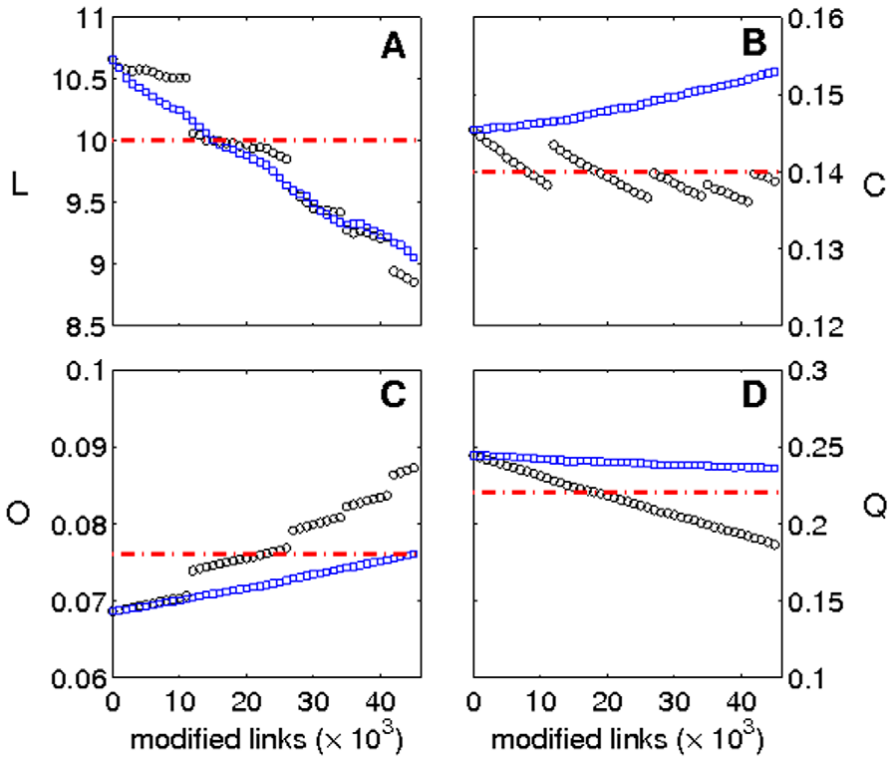
Modeling the disease. Evolution of network parameters [shortest path (A), clustering (B), outreach (C) and modularity (D)] as the number of impaired links increases. Red dashed lines are the mean values of the MCI group. Blue squares correspond to 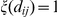 and black circles to 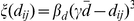. Parameters used in the simulations are given in Fig. 5 caption.

Finally, Fig. 6 shows the numerical results of the evolution of four network parameters (shortest path 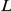, clustering coefficient 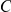, outreach 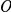 and modularity 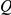) as the disease progresses starting from a healthy brain. We consider two different scenarios, one without the length influence 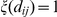 (blue squares) and other with 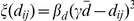 (black circles), with 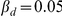 and 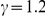 (other parameters are given in the caption of Fig. 5). In both cases, network parameters evolve in the direction of the MCI values (red dashed lines), with the only exception of clustering in absence of length dependence. With regard to the outreach 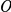 and modularity 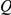, it is worth mentioning that the increase of weights at the long-range connections [i.e., 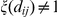] accelerates the process of the network deterioration.

## Discussion

The effect of MCI on brain networks dynamics is related to a group of phenomena that are hallmarks of an atypical network functioning. The relevant difference between healthy and MCI subjects is the increase in synchronized activity between brain areas. The enhancement in overall synchrony is reflected as an increase of average connectivity 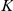 in functional networks and a reduction in the average distance between nodes 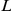. The second difference is that the increase in correlation is associated with an evolution towards random structures, as reflected by the normalized network parameters 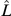, 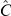 and 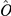, which are in all cases closer to unity. Despite the existence of an underlying random process, the increase in outreach 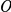 is much higher than it would be expected after a random reorganization of the network and indicates that the increase in synchronization is more frequent for long-range connections. This third difference plays a crucial role in the energetic cost, since patients suffering from MCI need to maintain correlations at long distances in order to successfully perform a memory task. An increase in the energetic cost with the same outcome indicates lower energetic efficiency. The network modularity 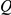 is dramatically affected according to all these observations. The evolution towards random topologies dilutes the identification of the network clusters, and the increase in weight of the long-range connections, makes network communities (lobes) more open. Both effects lead to a less modular network and break the subtle balance between segregation and integration processes. The conclusion is that, in order to compensate for the loss of the segregation and integration balance, MCI subjects tend to increase their long range synchronization which could be underlying the increased blood flow showed in fMRI studies during memory task [13].

Another indication that this synchronization profile might be related to a compensatory effort is the fact that the main differences between the control and MCI subjects are observed in the alpha band (see File S1 for details). This frequency band has been previously related with working memory task and its connectivity values are modulated by memory load [1]. Thus, the relation between the alpha band and working memory suggests that the increase in long range coordination showed by the MCI subjects might be revealing a reorganization of the network dynamics to compensate for the physiological malfunctioning associated with this neurological condition. Interestingly, MCI seems to share some of the neuropathophysiological characteristics of AD [31]. Examples include neurofibrilary tangles, which affect communication, the loss of synaptic contacts or the accumulation of the beta-amyloid protein which tend to happen in the associative cortex such as the temporal or the parietal lobes in both AD and MCI patients [7], [12], [32]. The parietal lobe has been recently associated with a hub, a highly connected region, in working memory tasks [1]. Thus, the physiological impairment of hubs could lead to the necessity of establishing a new configuration based on long distance connections to compensate for the lack of a centre which facilitates information communication.

Next, we developed a minimal network evolutionary model trying to capture the main signatures of MCI. The model shows that network parameters evolve in accordance with the observations, and allows one to understand how the progression of the disease could take place. Thus, as the functional network of a subject that is developing MCI increases its long distance connectivity, it is progressively mirroring the MCI network. The results suggest than an evaluation should be made on normal elderly subjects with subjective memory complaints (since some of them develop an objective cognitive impairment) in order to see if this tendency of communication based on long distance connections could be ultimately assessed as an early hallmark of cognitive impairment.

Many spatially distant, but functionally integrated functional networks have been described with fMRI and fcMRI analyses [33]–[35]. The results obtained in these distinct, but distributed, functional networks are compatible with our outcomes, although the type of analysis performed in each study is different (frequency domain in MEG versus blood flow in fMRI). The increase in the intralobe connections far from indicating a breakdown of integrated distributed networks (fMRI), are compatible with the integration of these functional networks, since interlobe connections overcome the increase of intralobe activity. The greater connectivity between anterior-posterior sites observed in the MCI group can be signaling the engagement of a dorsal fronto-parietal attentional network [36] which might reflect the greater executive/attentional resources that are necessary in order to accomplish the task for this group [37]. In fact, both techniques, MEG and fMRI, are adding complementary information pointing in the direction of a higher energetic cost in MCI subjects than in controls to perform the same memory task.

Finally, it is interesting to highlight the differences between the findings on MCI and Alzheimer disease (AD), since patients suffering from MCI are prone to develop AD. In both conditions, the distortion of the functional network is related to an evolution towards random structures, as indicated by a clustering coefficient and shortest path length that is closer to the random configuration. Both results are in accordance with the influence of aging in the increase of the network entropy, a concept recently formulated by Drachman [38]. Interestingly, the appearance of MCI is related to an increase of the connections in the network, contrary to what is observed in AD. Thus, MCI patients that evolve to Alzheimer's Disease must show, at some point, a sudden decrease in the synchronization of their functional networks. In this sense, forthcoming experiments should address whether connections which increase in value in MCI patients are later the ones that suffer the largest decrease in efficiency when the patient develops AD.

## Supporting Information

File S1 **Supporting Information.**
(PDF)
