# Supplementary material for: Reorganization of Functional Networks in Mild Cognitive Impairment
Source: PLoS One. 2011 May 23;6(5):e19584. doi: 10.1371/journal.pone.0019584 (PMC3100302; doi:10.1371/journal.pone.0019584)
Supplement: File S1 — Supporting Information. (PDF) [file pone.0019584.s001.pdf]

Reorganization of functional networks in mild cognitive impairment

(Supporting Information)

Javier M. Buldú, Ricardo Bajo, Fernando Maestú, Nazareth Castellanos,
Inmaculada Leyva, Pablo Gil, Irene Sendiña-Nadal, Juan A. Almendral,
Angel Nevado, Francisco del Pozo, and Stefano Boccaletti.

April 26, 2011

This document contains additional information and completes the results presented in the paper "Reorganization of functional networks in mild cognitive impairment" by Buldú et al. First, we show the variation of the network topological parameters at the six frequency bands defined in the main text (S1). Second, we include a Figure with the statistical significance of the parameter variation at the full bandwidth signal (S2). Next, we plot the position of each node at the phase space given by the participation coefficient p_i and the within-module-degree z_i (S3). This Figure, which complements Fig. 4 of the main document, allows to understand what nodes are more relevant inside each lobe and what nodes behave as connectors between lobes. In addition, we can observe what are the quantitative differences between the control group and the MCI group (Fig. S2, bottom plot). Finally, in the Materials and Methods section (S4), we give a detailed description of: a) the procedure followed during the selection of patients and controls, b) the memory task, c) the magnetoencephalographic recordings and d) the signal analysis.

S1 Variation of network parameters and frequency band analysis

Table S1 summarizes the results (average value and error of the mean) obtained for each group along with the percentage of variation from the control group ($\% = \frac{X_{MCI}-X_{cont}}{X_{cont}}$). Five frequency bands [α_1 : $(8-11)$Hz, α_2 : $(11-14)$Hz, β_1 : $(14-25)$Hz, β_2 : $(25-35)$Hz, γ : $(35-45)$Hz] were considered. In all cases, differences between groups are better observed at the broadband signal. When signal is split into frequency bands, the alpha band is the one showing more differences between controls and MCI patients. Changes over 5% are highlighted in colors (blue indicating an increase and red a decrease).

Table S1: Summary of the average network parameters for the control and MCI groups and their percentage of variation. Definition of parameters: K, average strength; C, clustering coefficient; O, outreach and Q, modularity. Symbol ˆ indicates normalization by the randomized version of the networks (see Section *Materials and Methods: Definition of Network Parameters* of the main document for details).

	Band	Control	MCI	MCI vs Control
K	all bands	12.1±0.5	14.0±0.5	+ 15.9 %
	α_1	16.2±0.5	17.1±0.6	+ 5.2 %
	α_2	15.5±0.5	15.9±0.5	+2.3%
	β_1	13.3±0.5	13.6±0.4	+1.8%
	β_2	12.8±0.3	13.3±0.3	+4.1%
	γ_1	15.9±0.7	16.5±0.5	+3.6%
L	all bands	10.7±0.3	10.09±0.22	-5.7%
	α_1	9.3±0.2	8.94±0.20	-4.0%
	α_2	9.6±0.3	9.34±0.24	-3.0%
	β_1	10.7±0.3	10.40±0.24	-2.5%
	β_2	11.5±0.3	11.2±0.3	-2.2%
	γ_1	10.2±0.5	9.7±0.3	-4.4%
$\hat{L}$	all bands	2.104±0.021	2.056±0.013	-2.3%
	α_1	1.916±0.019	1.876±0.020	-2.1%
	α_2	1.921±0.018	1.906±0.021	-0.8%
	β_1	2.049±0.019	2.036±0.015	-0.6%

Table S1. (Continued)

	Band	Control	MCI	MCI vs Control
	β_2	1.949±0.022	1.917±0.017	-1.7%
	γ_1	1.75±0.03	1.694±0.024	-3.2%
$C(\times 10^{-1})$	all bands	1.42±0.04	1.42±0.03	+0.1%
	α_1	1.41±0.04	1.46±0.05	+3.5%
	α_2	1.37±0.04	1.41±0.05	+2.6%
	β_1	1.31±0.04	1.34±0.04	+2.1%
	β_2	1.18±0.03	1.19±0.03	+1.0%
	γ_1	1.31±0.06	1.32±0.03	+0.2%
$\hat{C}$	all bands	1.76±0.05	1.52±0.05	-13.6%
	α_1	1.286±0.017	1.261±0.016	-1.9%
	α_2	1.307±0.013	1.305±0.012	-0.1%
	β_1	1.448±0.023	1.448±0.015	+0.0%
	β_2	1.353±0.021	1.313±0.009	-3.0%
	γ_1	1.220±0.017	1.177±0.012	-3.5%
$O(\times 10^{-2})$	all bands	6.1±0.4	7.6±0.4	+23.4%
	α_1	9.6±0.4	10.2±0.4	+6.3%
	α_2	9.1±0.3	9.2±0.3	+1.5%
	β_1	7.4±0.3	7.47±0.22	+0.8%
	β_2	7.36±0.19	7.73±0.21	+4.9%
	γ_1	9.9±0.5	10.4±0.4	+4.9%
$\hat{O}(\times 10^{-1})$	all bands	6.32±0.15	6.74±0.12	+6.7%
	α_1	7.38±0.08	7.49±0.05	+1.4%
	α_2	7.36±0.06	7.31±0.04	-0.6%
	β_1	6.96±0.08	6.90±0.04	-0.8%
	β_2	7.23±0.09	7.27±0.04	+0.6%
	γ_1	7.78±0.11	7.89±0.06	+1.4%
$Q(\times 10^{-1})$	all bands	2.57±0.09	2.22±0.08	-13.5%
	α_1	1.73±0.04	1.64±0.03	-5.3%
	α_2	1.75±0.03	1.75±0.02	+0.3%
	β_1	2.09±0.04	2.06±0.03	-1.2%
	β_2	1.97±0.04	1.90±0.03	-3.6%
	γ_1	1.65±0.05	1.54±0.04	-6.4%

S2 Statistical analysis

In Figure S1 we have plot the percentage of variation of the topological parameters for the full bandwidth (8-45 Hz), together with its statistical significance. Circles correspond to a $p < 0.03$ and stars to $p < 0.01$. Note that outreach is the parameter which is modified the most (and in addition $p_{outreach} < 0.01$), which indicates that it is the most suitable parameter in order to differentiate between healthy individuals and patients with MCI.

S3 Community structure and roles

Figure S2 complements Fig. 4 of the main document, which shows changes in the roles played by the main nodes of the network in the intra and inter lobular connections. We use the node characterization proposed by Guimerà et al. [1] that classifies the role of the nodes with regard to the function that they are playing inside and outside of their communities. Two parameters are calculated, the within-module degree z_i (also known as *z-score*) and the participation coefficient p_i. The first parameter computes the importance of the node inside its community and it is defined as:

$$z_i = \frac{k_i - \bar{k}_{c_i}}{\sigma_{k_{c_i}}} \tag{1}$$

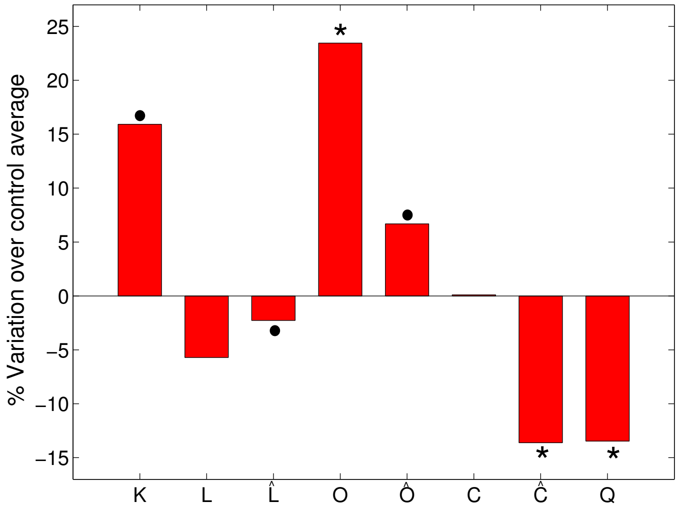

Figure S1: Percentage of variation of the average degree K, average shortest path L and its normalized value $\hat{L} = \frac{L}{L_{ran}}$, network outreach O and normalized outreach $\hat{O} = \frac{O}{O_{ran}}$, clustering C and normalized clustering $\hat{C} = \frac{C}{C_{ran}}$ and network modularity Q. Circles (•) correspond to $p < 0.03$ and stars (*) to $p < 0.01$, specifically: K ($p = 0.018$), $\hat{L}_z$ ($p = 0.025$), O ($p = 0.007$), $\hat{O}$ ($p = 0.027$), $\hat{C}$ ($p = 0.002$) and Q ($p = 0.0033$) .

where k_i and c_i are, respectively, the degree and the community c_i of the node i, $\bar{k}_{c_i}$ is the mean degree of the community c_i and $\sigma_{k_{ci}}$ is the standard deviation of k in c_i. On the other hand, the participation coefficient p_i indicates how connections of node i are distributed among the existing communities:

$$p_i = 1 - \sum_{c=i}^{N_c} \left(\frac{k_{c_i}}{k_i} \right)^2 \tag{2}$$

where k_{c_i} is the number of connections between node i and community c_i and N_c is the total number of communities. The participation coefficient is zero when all links of a node are inside its own community and close to one when they are distributed among all modules of the network. In Fig. S2(A) we have plotted the $[p_i, z_i]$ phase space of all nodes in the network for the control group. We can observe that, during a memory task, most participating nodes (high p_i) are located over the two frontal lobes (blue and black circles), while nodes with higher relevance (i.e., those with higher z_i) are located over the occipital lobe. Figure S2(B) shows the variation of both parameters in the MCI group (MCI minus control). In accordance with previous results, the participation coefficient increases in the majority of nodes, since connections between lobes are strengthen over the whole network. Regarding the within-module degree, we observe both positive and negative changes, which indicates a generalized reorganization inside each lobe.

S4 Supplementary materials and methods

Thirty-eight right handed, elderly participants recruited from the Geriatric Unit of the Hospital Universitario San Carlos (Madrid) participated in the study. Participants were divided into two groups based on their clinical profiles: nineteen participants were classified as multi-domain MCI patients, and the other nineteen as healthy control volunteers. MCI diagnosis was established according to the criteria proposed by Petersen [2]. Nineteen age-matched, healthy elderly volunteers, without memory complaints consented to participate in the study. Patients and controls underwent a neuropsychological assessment, in order to establish their cognitive status in multiple cognitive functions. Specifically, memory impairment was assessed by the Logical Memory (immediate and delayed) from the Wechsler Memory Scale-III-R. Two scales of cognitive and functional status were also applied: the Spanish version of the Mini Mental State Examination (MMSE) [3], and the Global Deterioration Scale/Functional Assessment Staging GDS/FAST [4]. In order to avoid possible differences due to the years of education, patients and controls were chosen so that the resulting average number of years of education was similar: 10 years for patients and 11 years for controls.

A modified version of the Sternberg's letter-probe task [5] was used as the memory test. A set of five letters was presented and participants were asked to keep the letters in mind. After the presentation of the five letter set, a series of single letters (500 ms in duration with a random ISI between 2–3 s) was presented one at a time, and the participants were asked to press a button with their right hand when a member of the previous set was detected. All participants completed a training session before the actual test, which did not start until participants demonstrated that they could remember the five-letter set. Letters were projected through a LCD video-projector (SONY VPL-X600E), situated outside of the shielded-room onto a series of in-room mirrors, the last of which was suspended approximately

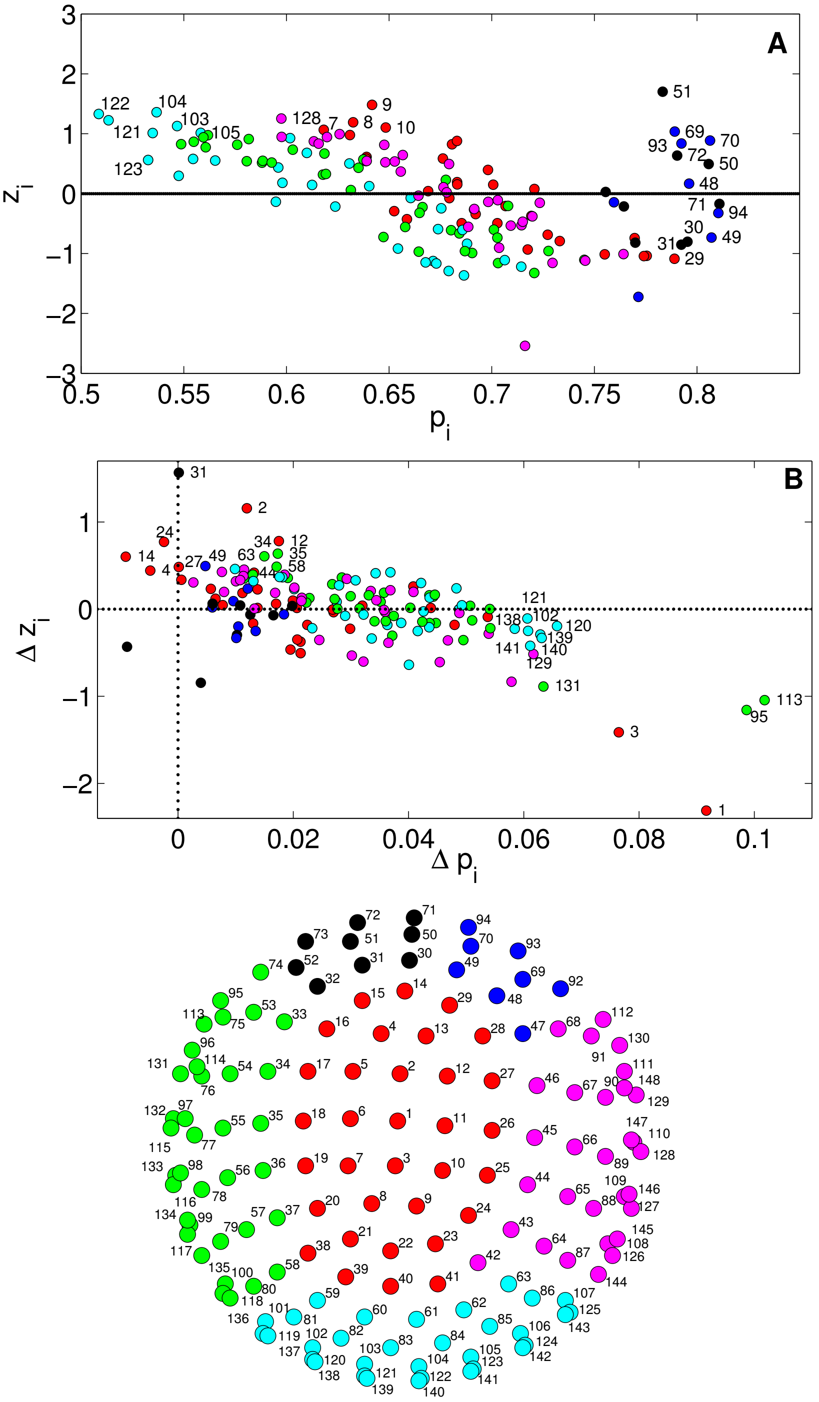

Figure S2: (A) Within-module degree z_i for each node in the network of the control group as a function of its corresponding participation coefficient p_i. Only the 13 nodes with the highest z_i and p_i are labelled. Data should be compared with Fig. 4 of the main document. (B) Within-module degree increment (Δz_i) between MCI and control group as a function of the participation coefficient increment (Δp_i) of each node. Again, only the 13 nodes with the highest differences are labelled. Bottom plot shows the Euclidean position of each node. Lobe color scheme: red-central, blue-frontal right, black-frontal left, magenta-temporal right, green-temporal left, and cyan-occipital.

1 meter above the participant's face. The letters subtended 1.8 and 3 degrees of horizontal and vertical visual angle respectively.

The MEG signal was recorded with a 254 Hz sampling frequency and a band pass of 0.5 to 50 Hz, using a 148-channel whole-head magnetometer (MAGNES 2500 WH, 4-D Neuroimaging) confined in a magnetically shielded room. An environmental noise reduction algorithm using reference channels at a distance from the MEG sensors was applied to the data. Thereafter, single trial epochs were visually inspected by an experienced investigator, and epochs containing visible blinks, eye movements or muscular artifacts were excluded from further analysis. Artifact-free epochs from each channel were then classified into four different categories, according to the subject's performance in the experiment:

hits, false alarms, correct rejections and omissions. Only hits were considered for further analysis because we were interested in evaluating the functional connectivity patterns which support recognition success. Thirty-five epochs were used to calculate Synchronization Likelihood (SL) between all pairs of nodes (electrodes) of each individual. This lower bound was determined by the participant with least epochs. To have an equal number of epochs across participants, thirty-five epochs were randomly chosen from each of the other participants.

In-house Fortran code was used to implement the SL algorithm as described by Stam et al. [6]. The SL algorithm was applied to the thirty-five extracted (artifact-free) one-second epochs of each subject. For each frequency band optimal SL parameter values were chosen according to Montez et al. [7]: lag $L = \frac{f_s}{3H_f}$, embedding dimension $M = \frac{3H_f}{L_f}$, theiler window $W_1 = 2L(M-1)$ and window length $W2 > \frac{10}{pref} + (W1 - 1)$, with parameter $pref$ below 0.05. Finally, f_s is the sampling rate, and H_f and L_f are the high and low frequency bounds, respectively.

As mentioned in the main document, the following frequency bands were considered: α_1 : $(8 - 11)$Hz, α_2 : $(11 - 14)$Hz, β_1 : $(14 - 25)$Hz, β_2 : $(25 - 35)$Hz, γ : $(35 - 45)$Hz. The SL index was not computed for bands under 8 Hz as the epoch length and sampling rate do not allow an accurate enough estimation [7]. All epochs were digitally filtered off-line at the above frequency bands. Subsequently, SL was calculated for each of the thirty-five one-second epochs of the $(148 \times 147)/2$ channel pairs, for each frequency band and the full-band signal, and for each subject (nineteen controls and nineteen patients).

References

[1] Guimerà R, Amaral LAN (2005) Functional cartography of complex metabolic networks. *Nature* 433:895–900.

[2] Petersen RC (2004) Mild cognitive impairment as a diagnostic entity. *J Intern Med* 256:183–194.

[3] Lobo A, Ezquerra J, Gomez BF, Sala JM, Seva DA (1979) Cognocitive mini-test (a simple practical test to detect intellectual changes in medical patients. *Actas Luso Esp Neurol Psiquiatr Cienc Afines* 7:189–202.

[4] Auer S, Reisberg B (1997) The GDS/FAST staging system *Int Psychogeriatr* 9:167–171.

[5] Maestu F, Fernandez A, Simos PG, Gil-Gregorio P, Amo C, Rodriguez R, Arrazola J, Ortiz T (2001) Spatio-temporal patterns of brain magnetic activity during a memory task in Alzheimer's disease. *Neuroreport* 12:3917–3922.

[6] Stam CJ, van Dijk BW (2002) Synchronization likelihood: an unbiased measure of generalized synchronization in multivariate data sets. *Physica D* 163:236–251.

[7] Montez T, Linkenkaer-Hansen K, van Dijk BW, Stam CJ (2006) Synchronization likelihood with explicit time-frequency priors. *Neuroimage* 33:1117–1125.
